# Initial investigation into patellofemoral morphology in hemophilic arthritis patients

**DOI:** 10.3389/fsurg.2024.1487156

**Published:** 2024-12-13

**Authors:** Haohao Wang, Rendong Jiang, Zhishang Dong, Dongyue Zhao, Jianli Zhao, Chao Shi, Zhen Yuan

**Affiliations:** Department of Orthopedics, The First Affiliated Hospital of Shandong First Medical University & Shandong Provincial Qianfoshan Hospital, Jinan, Shandong, China

**Keywords:** hemophilic arthropathy, patellar morphology, ligaments, clinical implications, total knee arthroplasty

## Abstract

**Background:**

Hemophilic arthritis (HA) is associated with significant changes in the morphology of mature knee joints due to abnormal growth plate development. Previous studies have established marked distinctions between the femur and tibia of subjects with Haemophilia and those with osteoarthritis (OA). This study explored the morphological characteristics of the patella and patellofemoral joint in subjects with Haemophilia. These findings can inform the design of knee joint prostheses tailored to this condition, improve the precision of total knee replacement surgery, and reduce postoperative knee pain and patellar dislocation.

**Methods:**

Before surgery, we conducted preoperative measurements of patellar length, patellar diagonal length, patellar ligament length, patellar width, patellar thickness, the INSALL index, the lateral patellofemoral angle, the trochlear groove angle,the patellar lateral displacement rate, and the patellofemoral index using lateral and axial x-ray images in 40 subjects with Haemophilia, 40 OA patients, and 40 normal individuals.

**Results:**

Significant statistical differences in certain morphological parameters were observed among the three groups of patients (*P* < 0.05). Compared with the OA and normal control groups, the HA group presented significant disparities in patellar thickness, patellar ligament length, the Insall ratio, the patellar lateral shift rate, the lateral patellar angle, and the patellofemoral index.

**Conclusion:**

Compared with OA and normal individuals, Subjects with Haemophilia presented with smaller and thinner patellae, more significant patellar ligament contracture, reduced patellar height, and more pronounced patellar dislocation. Consequently, during total knee arthroplasty, we lean toward patellar reshaping in subjects with Haemophilia, exercise caution when considering patellar replacement, and, for those with severe preoperative patellar dislocation, perform intraoperative lateral retinacular release.

## Introduction

Hemophilia is a hereditary bleeding disorder caused by a deficiency of clotting factors and can be classified into hemophilia A (factor VIII deficiency) and hemophilia B (factor IX deficiency). Patients with hemophilia often experience bleeding from a young age, primarily in joints, muscles, and deep tissues, with a pronounced impact on weight-bearing large joints (1).

Hemophilic arthropathy (HA) is one of the major complications of hemophilia. Its pathogenesis involves spontaneous intra-articular bleeding and recurrent hematomas within the joints, leading to joint contractures, deformities, and pain. This ultimately results in marked limitations in joint function or disability, severely impacting patients’ quality of life. The most commonly affected joints, in descending order of occurrence, are the knees, elbows, and ankle joints (2). The typical pathological features of HA include synovitis, joint cartilage damage, and subchondral bone deterioration.

On the one hand, even though prophylactic administration of factor VIII or factor IX can decrease the incidence of joint bleeding in subjects with Haemophilia, they may still experience sudden joint bleeding. Studies have shown that more than 90% of prophylactic patients develop chronic joint changes in at least one joint before the age of 40 (3–6). If patients receive only on-demand treatment, severe subjects with Haemophilia may have a life expectancy of just 20 years, and their chances of requiring orthopedic surgery significantly increase (4, 7).

**Figure 1:**
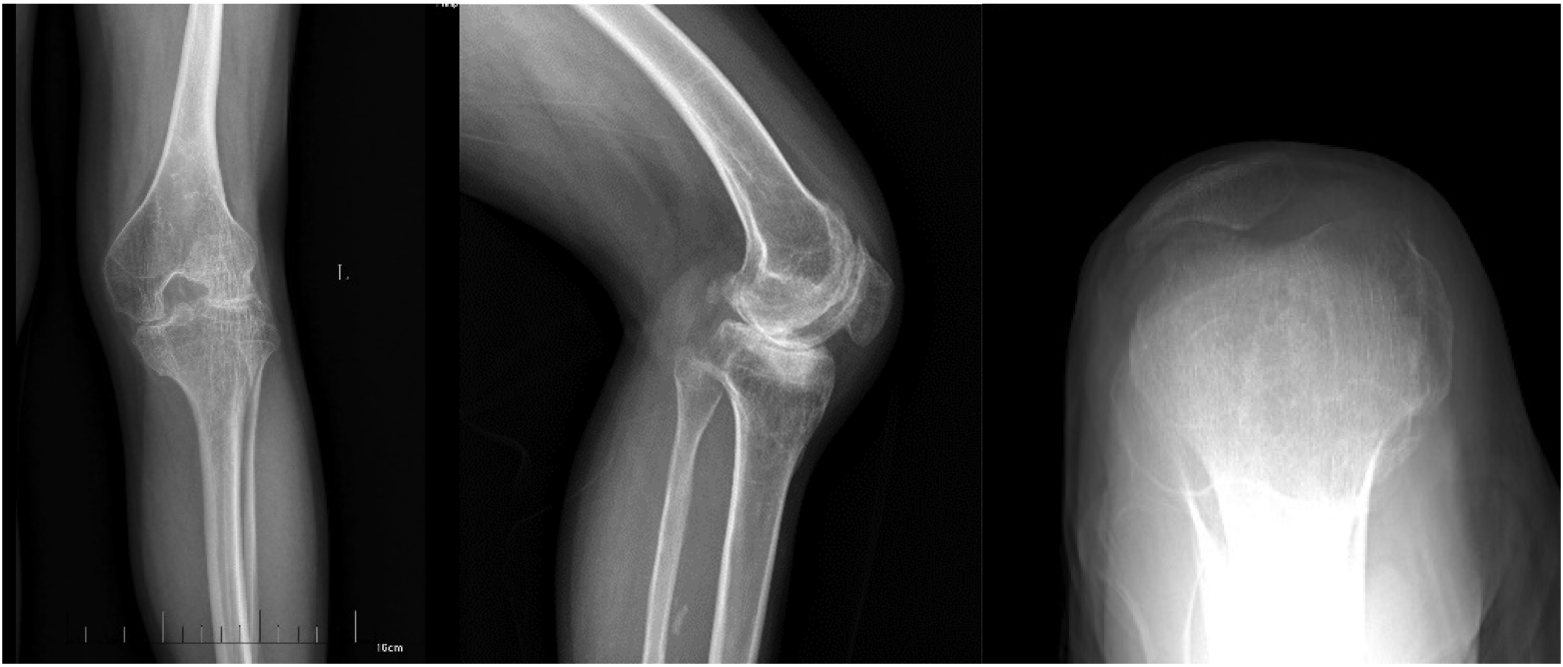
Radiography of a patient with hemophilic knee arthritis.

Total knee arthroplasty enhances the quality of life in late-stage subjects with Haemophilia and enjoys extensive clinical application. However, individuals with HA encounter numerous challenges and complications during TKA, primarily attributable to early disease onset and prolonged disease duration. Thus far, a comprehensive investigation into the patellar morphology of subjects with Haemophilia remains lacking. Gao et al. discovered through imaging measurements that subjects with Haemophilia exhibit larger intercondylar notches and medial condyles compared to osteoarthritis (OA) patients (8), and Feng et al. similarly observed through imaging measurements that the patellar tendon length in subjects with Haemophilia is notably shorter than in OA patients (9). Nevertheless, none of those mentioned above studies conducted measurements of patellar morphology or made comparisons with that of individuals without hemophilia. Several studies have documented distinctive radiological alterations in hemophilia-associated knee arthropathy: a prominent intercondylar notch; early cessation of patellar growth, resulting in a shortened longitudinal diameter and a relatively increased transverse diameter, giving rise to a square-shaped patella; and the potential presence of irregular sclerosis and damage at the posterior edge of the patella (10) (Figure 1).

Due to the developmental abnormalities of the patella, whether a patellar replacement should be routinely performed in HA, like in OA, needs further consideration. Research on patellar replacement in subjects with Haemophilia undergoing TKA is limited. Therefore, this work aims to explore the morphological characteristics of the patella and patellar groove in subjects with Haemophilia, perform imaging measurements, investigate their alterations, deepen our understanding of joint pathology in subjects with Haemophilia, and optimize the TKA technique for knee joint changes. This will lead to more satisfactory outcomes for subjects with Haemophilia after TKA, potentially aid in designing knee prostheses suitable for this condition, improve the accuracy of total knee replacement surgery, and provide a theoretical basis for avoiding postoperative knee pain and patellar dislocation.

## Materials and methods

A retrospective analysis of the medical records of 40 patients with HA who underwent unilateral TKA at Qianfoshan Hospital, Shandong Province, from February 2018 to March 2022 was conducted. This study received approval from the Research Ethics Committee of Qianfoshan Hospital, Shandong Province. Informed consent was obtained from all the subjects or their legal guardian(s).

Inclusion criteria: clinical diagnosis of HA; no history of lower limb fractures (including patella, femur, tibia); complete patient demographic information; absence of coagulation factor inhibitors; absence of noticeable pseudotumor in subjects with Haemophilia, with evident indications for joint replacement.

Exclusion criteria: inability of the knee joint to flex to 30°; history of previous lower limb fractures; history of lower limb bone tumors; non-standard image capture. Among the 40 screened subjects with Haemophilia, the ages ranged from 23 to 43, with radiographic examinations indicating Arnold-Hilgartner IV–V grades.

For comparative analysis, we matched subjects with Haemophilia based on age, gender, height, weight, and BMI with patients who underwent knee joint lateral and axial x-rays for non-bone joint disorders such as OA, osteoporosis, and fractures within the past four years at our hospital, forming the negative control group. We also selected patients with Kellgren-Lawrence (KL) radiographic grading of 3–4 for bone joint arthritis who met the inclusion and exclusion criteria as the positive control group. In the end, we included 40 cases in the hemophilic group, 40 cases in the arthritis group, and 40 cases in the normal group. Population demographics are detailed in Table 1.

**Table 1:** Demographic data.

Patient demographics	HA group (*n* = 40) mean ± SD	OA group (*n* = 40) mean ± SD	Normal group (*n* = 40) mean ± SD (range)	*F*	*P*
Age (years)	31.58 ± 5.39	67.35 ± 5.82^a^	32.33 ± 7.23^b^	434.609	<0.05
Height (m)	1.72 ± 0.02	1.73 ± 0.02	1.73 ± 0.04	1.609	0.205
Weight (kg)	68.76 ± 6.8	70.58 ± 5.2	71.12 ± 5.4	1.865	0.159
BMI (kg/m^2^)	23.31 ± 2.3	23.70 ± 1.8	23.80 ± 2.3	0.595	0.553

^a^ Indicates comparisons with HA, where the *p*-value is less than 0.05.

^b^ Indicates comparisons with OA, where the *p*-value is less than 0.05. All pairwise comparisons underwent Bonferroni correction.

**Figure 2:**
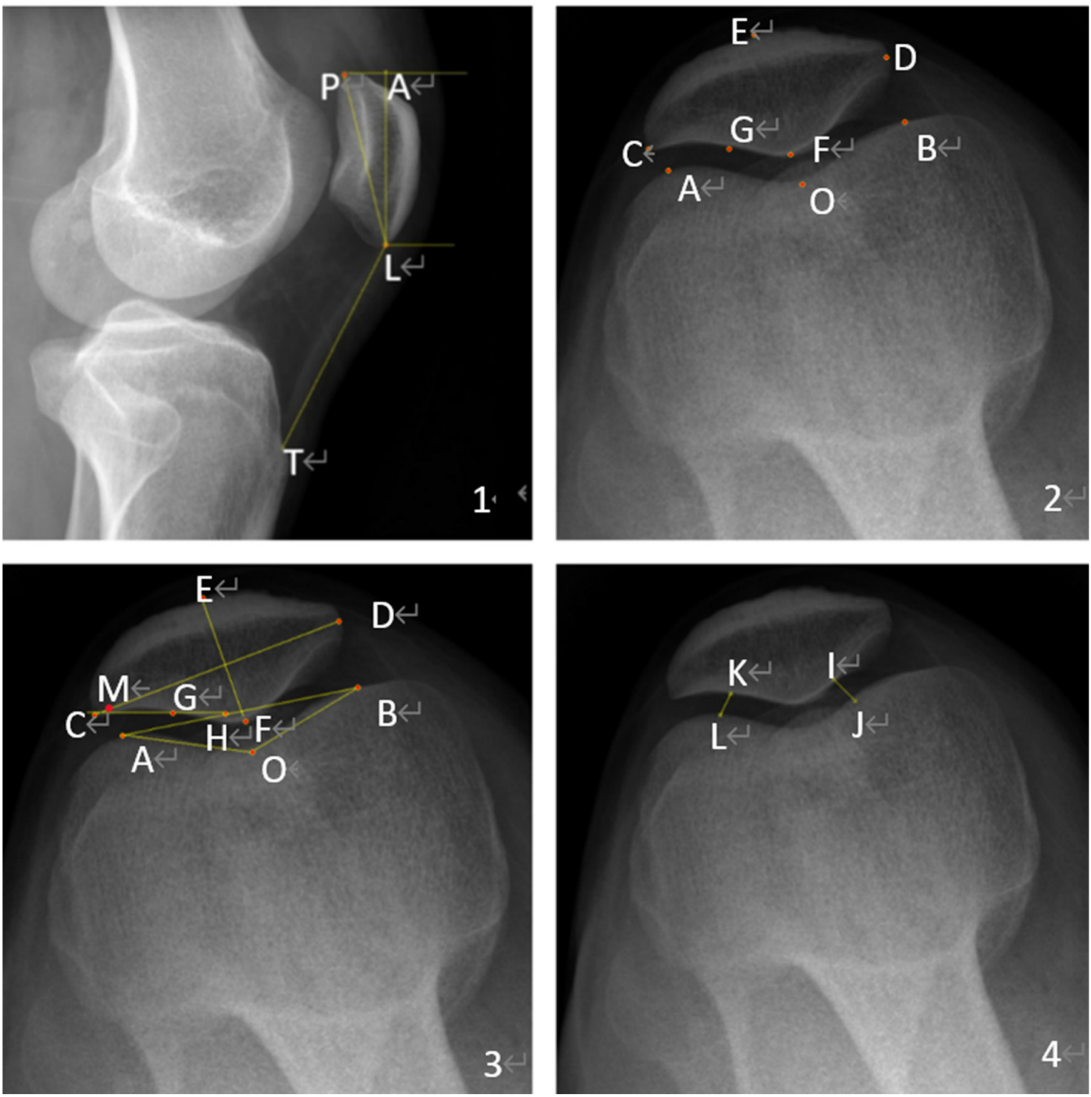
Measurement parameters for the patellofemoral. 1. X-ray of the lateral patellofemoral. 2. Location of landmarks on an axial x-ray image. P superior pole of the patella. L Inferior pole of patella. T Tibial tuberosity inferior pole. The Insall-Salvati ratio = LT/LP, and the patellar length = AL (Figure 2.1). The highest point of the lateral condyle. B Highest point of the medial condyle. C Outermost point of patella. D Most medial point of patella. E Anterior cortex of the patella. F Median cartilaginous ridge of patella. G Tangent point of the lateral articular surface of the patella (Figure 2.2). H The intersection of the lateral patella's articular surface and the femoral internal and external condyles line. M is the point at which the highest point of the lateral epicondyle of the femur intersects with the width of the patella. O Most distal point at the bottom of the trochlear groove Patellar width = CD, Patella thickness = EF. Groove angle = angle AOB. Patellar displacement rate = CM/CD. Lateral patellofemoral angle = angle GHA (Figure 2.3). IJ, KL, the patellofemoral joint's internal and lateral spaces, are connected in the shortest distance. Patellofemoral index = IJ/KL (Figure 2.4).

Standard lateral and patellofemoral joint axial x-rays were captured using the Philips Medical Systems (DMC Gmbh, Hamburg/GERMANY, X-RAY TUBE HOUSING ASSEMBLY). For the lateral view, the patient lay supine with the knee flexed at 30° to relax the quadriceps femoris. The imaging plate was positioned on the outer side of the knee, and the x-ray was taken from the inner side of the knee. For the axial view of the patella, the patient sat on the examination bed with their feet close to the edge. The x-ray was aligned parallel to the anterior edge of the tibia, with the knee flexed at 20°. The patient positioned the cassette against their thigh, ensuring a 90° angle with the beam. The raw data were then imported into Digimizer software. The following morphological parameters were measured sequentially: patellar length, diagonal length of the patella, length of the patellar ligament, patellar width, patellar thickness, Insall index, lateral patellofemoral angle, trochlear angle, patellar lateral shift, and patellofemoral index (refer to Figure 2). Following the same measurement method, one physician independently took three measurements, and the average value was recorded to ensure data objectivity.

### Statistical analysis

All the statistical analyses were conducted using SPSS 26.0 software (IBM, Inc., Armonk, NY, USA). We conducted normality tests to determine whether the three sample populations followed a normal distribution. One-way analysis of variance was applied for normally distributed data, while the Mann-Whitney U test was employed for non-normally distributed data. A significance level of *P* < 0.05 indicated statistical significance.

## Results

No significant differences were observed in gender, age, weight, height, and BMI between the HA and negative control groups (*P* > 0.05). However, the HA group and the negative control group (normal group) differed significantly from the OA group only in terms of age (*P* < 0.05) (Table 1).

Significant differences were found in patellar ligament length, patellar transverse diameter, patellar thickness, patellar width, and the INSALL index when comparing the HA group to the OA group and normal individuals (*P* < 0.05) (Table 2).

**Table 2:** Patellar morphology.

Morphological variables	HA group (*n* = 40) mean ± SD (range)	OA group (*n* = 40) mean ± SD (range)	Normal group (*n* = 40) mean ± SD (range)	*F*	*P*
Superior articular surface interior pole (SI) (cm)	4.44 ± 0.34	4.53 ± 0.25	4.31 ± 0.28^b^	5.37	0.05
Tibial tuberosity inferior pole (TI) (cm)	3.93 ± 0.31	5.18 ± 0.58^a^	5.08 ± 0.39^a^	17.56	0.05
Patellar width (cm)	4.76 ± 0.24	4.48 ± 0.34^a^	4.26 ± 0.32^a^	7.827	0.05
Patellar thickness (cm)	1.67 ± 0.27	2.35 ± 0.37^a^	2.24 ± 0.15^a^	5.56	0.05
Insall-Salvati ratio (TI/SI)	0.88 ± 0.06	1.15 ± 0.15^a^	1.18 ± 0.11^a^	5.457	0.05

^a^ Indicates comparisons with HA, where the *p*-value is less than 0.05.

^b^ Indicates comparisons with OA, where the *p*-value is less than 0.05. All pairwise comparisons underwent Bonferroni correction.

There were statistically significant differences in the HA group's groove angle, lateral patellofemoral angle, patellar displacement rate, and patellofemoral index compared to the OA group and the normal control group (*P* < 0.05), there was no statistically significant differences in patellar length among the three groups (*P* > 0.05) (Table 3).

**Table 3:** Patellofemoral morphology.

	HA group (*n* = 40)	OA group (*n* = 40)	Normal group (*n* = 40)	*H*	*P*
Patellar length	4.27 (4.13, 4.51)	4.26 (4.13, 4.62)	4.17 (4.03, 4.40)	4.931	>0.05
Groove angle	129.34 (115.89, 136.41)	138.22 (134.36, 141.73)^a^	137.00 (134.36, 141.73)^a^	18.817	<0.05
Lateral patellofemoral angle	13.61 (12.19, 15.68)	18.18 (18.56, 19.36)^a^	16.05 (13.10, 20.27^a^	22.461	<0.05
Patellar displacement rate	0.21 (0.19, 0.27)	0.16 (0.11, 0.19)^a^	0.15 (0.12, 0.19)^a^	36.082	<0.05
Patellofemoral index	1.65 (1.31, 2.45)	1.13 (0.95, 1.33)^a^	1.17 (1.00, 1.40)^a^	36.569	<0.05

^a^ Indicates comparisons with HA, where the *p*-value is less than 0.05.

^b^ Indicates comparisons with OA, where the *p*-value is less than 0.05. All pairwise comparisons underwent Bonferroni correction.

## Discussion

Our study revealed statistically significant differences in the patellar and trochlear morphological parameters between subjects with Haemophilia and the OA and negative control groups (normal group). When examined from the knee joint axis and lateral x-ray perspective, subjects with Haemophilia presented thinner patellar thickness, lower patellar height, and more pronounced patellar tilt and dislocation. In a recent study, Arman Vahabi (11) and colleagues utilized MRI to assess patellofemoral morphology in patients with hemophilic arthritis (HA) as well as in healthy individuals. These findings revealed significant morphological changes in the patellofemoral joint of subjects with Haemophilia, such as a reduced patellar size, decreased intercondylar depth, shortened lateral condyle length, and narrowed trochlear groove. These observations further substantiate the conclusions drawn from this research.

### Patellar thickness

Our study revealed that the patellar thickness in subjects with Haemophilia is only 16.7 ± 2.7 mm (12). Our colleagues conducted measurements on subjects with Haemophilia undergoing TKA, and their results revealed an average patellar thickness of 16.3 ± 0.4 mm, which further supports our findings that compared with OA patients and the negative controls, Subjects with Haemophilia have relatively thinner patellae.

The key to successful patellar replacement is ensuring that the patella has adequate thickness, with a postosteotomy requirement of 15 mm (13). When the patella is excessively thin, the risk of fracture increases under substantial pressure, leading to a significantly greater probability of postoperative prosthesis loosening. Extensive bone loss, late-stage osteoporosis, and inflammatory conditions are all considered.

In this study, subjects with Haemophilia underwent a standard midline incision with a medial parapatellar arthrotomy, patellar valgus during surgery, V-Y quadriceps myoplasty if extension contractures, and then valgus patella. We performed extensive synovectomy and we preferred to release the posterior capsule and soft tissue rather than additional osteotomy to treat minor deformities.

To date, the decision to include patellar replacement in total knee arthroplasty (TKA) remains a contentious issue within the academic community, and there is no universally accepted standardized approach for prosthetics in Subjects with Haemophilia. Thoroughly analyzing patellofemoral morphological abnormalities and addressing them during arthroplasty are crucial for preventing postoperative patellofemoral complications. There is currently long-term experience published on TKA for hemophilic arthritis. Most available reports describe the use of standard cobalt-chromium femoral components and titanium tibial plates, materials commonly selected for elderly patients globally. As mentioned, Subjects with Haemophilia s typically undergo TKA at a younger or adult age, making the selection of high-performance implants essential. Christian Carulli et al. (14) utilized specific biomaterials, including an oxidized zirconium femoral component, a titanium tibial component, and highly crosslinked polyethylene (PE). Thelong-term follow-up indicated a high survival rate for these implants.

To date, relatively few reports exist on whether patellar replacement should be performed during TKA in subjects with Haemophilia. We attempted to address this issue by studying patients with rheumatoid arthropathy (RA) who share certain similarities with subjects with Haemophilia. Compared with OA patients, both RA and HA patients exhibit significant bone loss and osteoporosis. Holt's (15) study on whether to perform patellar replacement should be performed in RA patients undergoing TKA showed that preserving the patella had highly satisfactory effects on postoperative function and anterior knee pain relief. RA and HA share common characteristics, including reduced periarticular bone mass, osteoporosis, subpar subchondral bone quality, and soft tissue deformities.

To summarize, we suggest that subjects with Haemophilia and arthritis should follow distinct surgical strategies. In the Chinese population, we prefer to perform patellar reshaping consisting of articular surface smoothing, osteophyte removal and patellar rim denervation during TKA in subjects with Haemophilia and approach patellar replacement cautiously to mitigate the risks associated with thinner patellae.

### Patellar height

Our study revealed that HA patients' patellar ligament length and Insall index were significantly shorter than those of OA patients and healthy individuals. The Insall index approached the low position of the patella, likely due to recurrent joint cavity bleeding and scar formation in subjects with Haemophilia, resulting in patellar ligament contraction.

The patellar tendon, a crucial component of the knee extension mechanism, influences the range of motion of the knee joint through changes in its length. Research on the ligaments of hemophilic patients is currently limited. Zheng et al. (16) reported that, compared with OA patients, HA patients have more frequent rupture sites in collagen and elastic fibers within their ligaments. In HA patients, repeated bleeding into the joint cavity results in patellar tendon contracture and cartilage damage. This contracture can lead to a lower patella, which increases the patellofemoral joint pressure and further exacerbates cartilage damage.

According to a biomechanical study of knee joints (17), a low patellar position shortens the knee extensor mechanism's lever arm, altering the quadriceps muscle's mechanical advantage and restricting knee joint flexion. Our clinical observations showed that subjects with Haemophilia exhibited significantly more quadriceps lag after TKA than OA patients. Extensive studies have indicated (18–21) that post-TKA, patellar height decreases in OA patients, and those with low patellar position have poor knee joint mobility. This might be due to intraoperative patellar flipping causing swelling and contraction of the patellar ligament and complete removal of the infrapatellar fat pad, leading to ischemic contraction of the patellar ligament. Our research confirmed that the preoperative patellar height in subjects with Haemophilia was lower than that in OA patients and healthy individuals. This finding explains the poorer outcomes of TKA in subjects with Haemophilia compared to OA patients. Therefore, when conducting TKA for subjects with Haemophilia, it is recommended to selectively preserve the infrapatellar fat pad to minimize the likelihood of postoperative patellar height reduction.

### Patellofemoral morphology

In this study, significant differences were observed in the trochlear angle, lateral patellar angle, patellar lateral shift rate, and patellar index between subjects with Haemophilia and both the OA and the negative control group. These findings provide evidence of distinct patellar morphological abnormalities in subjects with Haemophilia, with a notable inclination towards dislocation.

Subjects with Haemophilia experience disease onset during childhood, characterized by recurrent erosion of cartilage and subchondral bone, which results in abnormalities in the epiphysis and metaphysis in adulthood. If the patella epiphysis is not spared from this pathological process, patellofemoral morphological changes in subjects with Haemophilia become nearly inevitable. Abnormal patellofemoral morphology will undoubtedly affect the relative relationship of the patellofemoral joint, subsequently influencing normal patellar tracking. Van Haver (22) discovered that during knee flexion and extension, patellofemoral joint pressure is distributed across different articular surfaces of the patella at varying flexion angles, preventing prolonged and excessive pressure stimuli that cause cartilage wear. We hypothesize that the patella exhibits lateral tilting and outward migration in subjects with Haemophilia. This significantly increases stress on the patellofemoral joint surfaces, while the contact area between the patella and femoral trochlea significantly decreases. Abnormal patellar development disrupts the balance of patellofemoral joint pressure distribution. The outward migration of the patella concentrates pressure on the lateral side of the patella and the later side of the trochlear surface, leading to excessive lateral patellar growth. This atypical pressure distribution may significantly contribute to patellofemoral morphological changes in subjects with Haemophilia.

During knee flexion, the patella glides from the patellar pouch to the intercondylar notch of the femur. In this process, the patella initially moves inward and then shifts outward in the coronal plane. This motion is not restricted only by the surrounding soft tissues. Nevertheless, it is also limited by anatomical structures, primarily the patella and trochlear structures, which oppose each other to maintain dynamic equilibrium. The morphological abnormalities in the patellofemoral joint of HA hinder the maintenance of this balance. Consequently, the patellar trajectory is altered, resulting in patellar tilt, lateral movement, or dislocation, in line with our clinical observations.

This study identified patellar subluxation in subjects with Haemophilia, possibly linked to contraction of the lateral patellar supporting band. Biomechanical studies (23) suggest that lateral supporting band release surgery can alleviate pressure on the lateral facet of the patella, thus relieving anterior knee pain. Extensive research has indicated that routine lateral supporting band release in patellar dislocation patients leads to improved knee joint functional scores (24–26),which is consistent with the lateral supporting band release performed in this study and provides theoretical support for our findings.

## Limitations

This study has several limitations. First, the number of cases was relatively limited, necessitating a larger sample size. Second, all measurement metrics in this study are limited to two-dimensional measurements and do not accurately reflect the complete relationship of the patellofemoral joint in subjects with Haemophilia. In future research, we will collect additional knee joint MRI data from subjects with Haemophilia to assess patellofemoral stability. Furthermore, all the numerical values were recorded by a single observer, introducing the possibility of assessor bias. Finally, it is essential to note that our findings are confined to theoretical research and lack support from clinical validation data.

## Advantages

In this study, we matched three groups of patients on the base of sex, height, weight, and BMI to reduce confounding factors, thereby enhancing the comparability between the experimental and control groups and minimizing the influence of individual variations. Additionally, we conducted a systematic analysis of the patellar and trochlear morphology in subjects with Haemophilia for the first time. This analysis should guide orthopedic surgeons in managing knee joints of subjects with Haemophilia with notable morphological changes in the patella and trochlea.

## Conclusions

Compared with patients with OA and individuals without joint conditions, those with HA presented smaller and thinner patellae, more pronounced patellar ligament contracture, reduced patellar height, and more significant lateral patellar dislocation. As a result, our preference during TKA in subjects with Haemophilia is to perform patellar reshaping and to approach patellar replacement cautiously. In cases of severe preoperative lateral patellar dislocation, intraoperative lateral patellar retinacular release is deemed necessary.

## Data Availability

The original contributions presented in the study are included in the article/Supplementary Material, further inquiries can be directed to the corresponding author.
